# Self-Assembled Biocompatible Fluorescent Nanoparticles for Bioimaging

**DOI:** 10.3389/fchem.2019.00168

**Published:** 2019-03-28

**Authors:** Valeria Caponetti, Jakub W. Trzcinski, Andrea Cantelli, Regina Tavano, Emanuele Papini, Fabrizio Mancin, Marco Montalti

**Affiliations:** ^1^Dipartimento di Chimica “Giacomo Ciamician”, Università di Bologna, Bologna, Italy; ^2^Dipartimento di Scienze Chimiche, Università di Padova, Padova, Italy; ^3^Dipartimento di Scienze Biomediche, Università di Padova, Padova, Italy

**Keywords:** fluorescence, fluorescent probes, ultra-bright, nanoparticles, nanoprecipitation, tracking, single particle imaging, bioimaging

## Abstract

Fluorescence is a powerful tool for mapping biological events in real-time with high spatial resolution. Ultra-bright probes are needed in order to achieve high sensitivity: these probes are typically obtained by gathering a huge number of fluorophores in a single nanoparticle (NP). Unfortunately this assembly produces quenching of the fluorescence because of short-range intermolecular interactions. Here we demonstrate that rational structural modification of a well-known molecular fluorophore N-(7-nitrobenz-2-oxa-1,3-diazol-4-yl) (NBD) produces fluorophores that self-assemble in nanoparticles in the biocompatible environment without any dramatic decrease of the fluorescence quantum yield. Most importantly, the resulting NP show, in an aqueous environment, a brightness which is more than six orders of magnitude higher than the molecular component in the organic solvent. Moreover, the NP are prepared by nanoprecipitation and they are stabilized only via non-covalent interaction, they are surprisingly stable and can be observed as individual bright spots freely diffusing in solution at a concentration as low as 1 nM. The suitability of the NP as biocompatible fluorescent probes was demonstrated in the case of HeLa cells by fluorescence confocal microscopy and MTS assays.

## Introduction

Fluorescence imaging is a not invasive, highly sensitive, technique that allows to investigate biological organisms with high tridimensional resolution in real time, by making use of suitable fluorescent contrast agents (Rio-Echevarria et al., 2011; Cauzzi et al., 2012; Chen et al., 2015; Grimm et al., 2015; Lee et al., 2015; Mei et al., 2015; Tang et al., 2015; Antaris et al., 2016; Proetto et al., 2016; Xu et al., 2016). Tailored fluorescent nanoparticles (NP) (Jiang et al., 2015; Ma et al., 2015; Pyo et al., 2015; Wolfbeis, 2015; Muller et al., 2018), promise to surpass conventional molecular probes as fluorescent markers especially as far as sensitivity is concerned: in fact, NPs potentially emit a much brighter signal, with respect to molecules, in the same excitation conditions. Nevertheless, achieving such an enhanced brightness (defined as B = εQY, where ε is the molar absorption coefficient of the NP and QY is fluorescence quantum yield of the NP) is still a challenge (Ow et al., 2005; Wu et al., 2006; Sun et al., 2010; Cho et al., 2011; Volkov et al., 2011; Trofymchuk et al., 2017; Melnychuk and Klymchenko, 2018).

Most of the NP proposed and applied for bio-imaging, in fact, result from the assembly of molecular fluorophores (MF) in an organized nanostructure. These assemblies may be stabilized: (i) via covalent bonds, hence by modifying the MF chemical structure with reactive terminal groups [e.g., alkoxysilanes (Rio-Echevarria et al., 2010; Rampazzo et al., 2011; Selvestrel et al., 2013), acrylates (Chen et al., 2009), or thiolates (Battistini et al., 2008; Bonacchi et al., 2016)] to form polymer/copolymer or by (ii) non-covalent interactions, that involve either the MF or additional groups specifically introduced in the structure to achieve supramolecular polymerization (Genin et al., 2014; Montalti et al., 2014; Reisch and Klymchenko, 2016; Faucon et al., 2017; Boucard et al., 2018). Control of size and size distribution is a critical issue in NP design and it can be achieved by exploiting surfactants or stabilizers as templates, hence molecules that are physically or chemically incorporated in the NP typically giving a compartmentalized structure (e.g., core-shell).

From the point of view of fluorescence brightness, the ability of NP to generate an intense fluorescence signal, even in the low intensity excitation regime, results from the co-presence of a high number of MF in each NP. Here, we report the preparation in a bio-compatible environment of NP with a diameter of about 90 nm containing as much as about 1 × 10^6^ MF/NP obtained by the self-assembling of new molecular fluorophores specifically designed to achieve highly bright NP.

We would like to stress that without a rational design, MF normally aggregate in highly densely packed NP undergoing strong fluorescence quenching, a process which reduces their QY to almost zero. This phenomenon known as aggregation caused quenching (ACQ) (Genovese et al., 2013), typically occurs in self-assembled multi-fluorophoric systems and it is the result of short-range interactions between MF in the NP.

ACQ is particularly severe in the case of actual contact between the MF, as it occurs when aggregates are formed, and it can be prevented, at least in part, by spacing the MF (Mak et al., 1999) by incorporating them in an inactive matrix (e.g., silica) as in typical dye-doped NP. A major drawback of this strategy, that becomes effective for NP with a dye/matrix ratio below ~1%, is a drastic reduction of the density of emitting molecules in the NP and hence of the ε of the NP with respect to matrix-free NP.

Here we describe the synthesis and the properties of a new family of MF, which are water insoluble and do not suffer from ACQ, and we demonstrate that a rational design of the molecular structure allows us to achieve a molecular unit that self-organizes in highly bright, and fluorescent NPs which are very stable in PBS (phosphate buffered saline) solution.

In designing the new MF, our attention was attracted by the N-(7-Nitrobenz-2-oxa-1,3-diazol-4-yl) (NBD) dye derivatives (Fery-Forgues et al., 1993; Mukherjee et al., 1994). This family of dyes has very interesting properties for imaging applications. In particular the synthetic precursor NBD chloride is quite inexpensive and it is easily conjugated with amine derivatives yielding fluorescent dyes featuring absorption and emission in the visible range, large Stokes shift and good quantum yields.

Unfortunately, emission of NBD derivative in the aggregated form is often very low and it is almost completely quenched in aqueous environment (Fery-Forgues et al., 1993). We reasoned that such a limitation could be overcome by the introduction of a triphenylphosphazene group in the NBD structure. Indeed such chemical modification, even if scarcely studied, has been reported to result, in the case of fluorescent dyes, into a red shift of both the absorption and luminescence maxima, an increase of the Stokes shift and of the luminescence intensity (Bodige et al., 1999; Nifant'ev et al., 2008; Joshi et al., 2014; Xu et al., 2015;Ragab et al., 2016).

Three different NBD-triarylphosphazene derivatives were prepared in high yields with straightforward procedures. The NP were then prepared via nanoprecipitation in the presence of Pluronic F127 as a stabilizer and they were characterized, from the photo-physical point of view, via UV-Vis absorption spectroscopy and steady-state and time resolved fluorescence spectroscopy. Although different surfactants have been proposed as stabilizer for nanoprecipitation we chose Pluronic F127 in virtue of its well-known biocompatibility (Pitto-Barry and Barry, 2014). The formation and stability of the NPs were demonstrated by dynamic light scattering (DLS) and transmission electron microscopy (TEM). Moreover, wide field fluorescence microscopy proved that these NP are stable at a concentration as low as 1 nM.

By comparing the NP to the molecular precursors, an increase of the brightness of about five order of magnitude, with respect to the fluorophore in organic solvent, could be estimated as a result of the self-assembly. A direct comparison with the fluorophore in aqueous medium was not possible because of the lack of solubility but making reference to an NBD water soluble derivative, we could appraise for the NP a brightness of more than six orders of magnitude higher. In order to demonstrate that these NPs were suitable for bio-imaging, they were incubated with living HeLa cells and their ability to label the cells was demonstrated via confocal scanning fluorescence microscopy. Finally, toxicity assays demonstrated the high biocompatibility of the NPs. We believe that these highly bright, functionalizable NPs are very promising platform for the design of new versatile multifunctional nanoprobes.

## Results and Discussion

### Photophysical Properties of the Molecules in Solution

**Figure 1:**
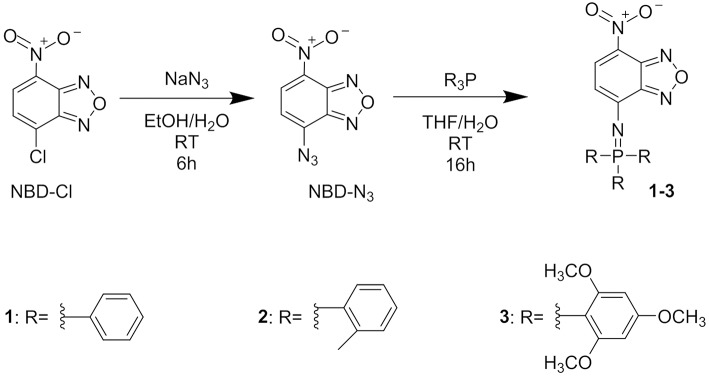
Chemical formula of compounds 1, 2, 3 and scheme of the synthetic reactions for their preparation.

Molecular fluorophores **1-3** are shown in Figure 1 and they were synthesized following the general reaction reported in Figure 1. Photophysical properties of molecules **1-3** where investigated in CH_2_Cl_2_ solution at the concentration 5 × 10^−5^ M and they are summarized in Table 1. In particular, the effect of the substituent on the phenyl ring was investigated. We would like to stress that fluorophores **1-3** are insoluble in water but that they can be dispersed in water in the form of NPs as discussed in the next section.

**Table 1:** Photophysical data of compounds 1, 2, 3, 4 and of 2NP and 3NP.

**Compounds**	**Solvents**	**λ_max, ass_ (nm)**	**ε_max_ (M^−1^cm^−1^)**	**λ_max, fluo_ (nm)**	**QY**	**<n>^b^**	**B^b^ (M^−1^cm^−1^)**	**<d>^b^ (nm)**
**1**	CH_2_Cl_2_	482	34,300	536	0.50	–	1.7 × 10^4^	–
**2**	CH_2_Cl_2_	488	32,600	526	0.65	–	2.1 × 10^4^	–
**3**	CH_2_Cl_2_	524	26,200	555	0.09	–	2.6 × 10^3^	–
**4^c^**	H_2_O	482	28,000	566	0.03	–	8.4 × 10^2^	–
**2NP**	H_2_O^a^	480	29,300	536	0.31	1.1 × 10^6^	1.0 × 10^10^	91 ± 13
**3NP**	H_2_O^a^	520	17,800	556	0.01	1.6 × 10^5^	2.8 × 10^7^	54 ± 9

a *PBS*.

b *Calculated from TEM*.

c *From Fery-Forgues et al. (1993)*.

**Figure 2:**
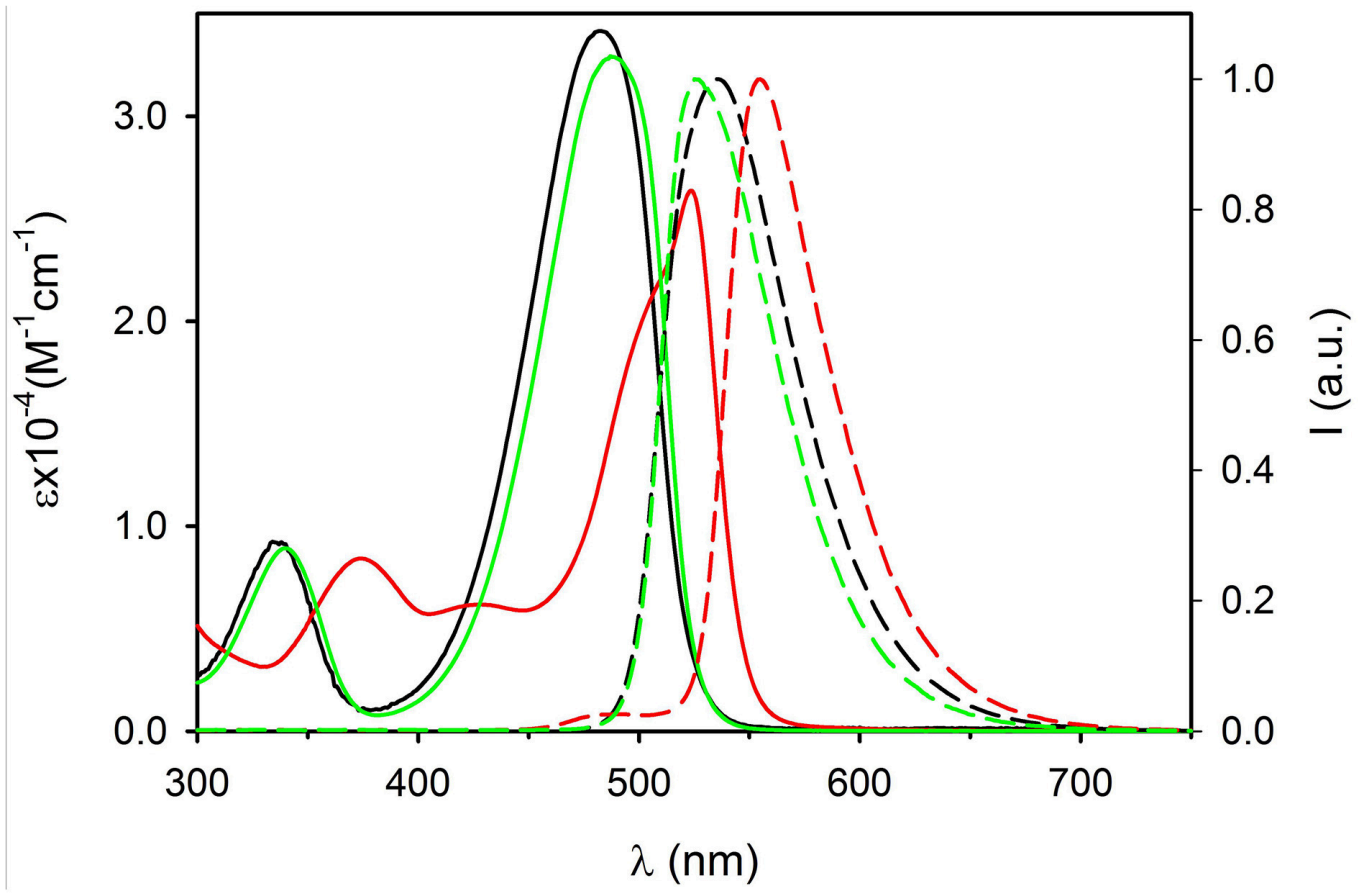
Absorption (continuous lines) and fluorescence spectra (dashed lines) of molecules 1 (black, λ_exc_ = 450 nm) 2 (green, λ_exc_ = 450 nm) and 3 (red, λ_exc_ = 490 nm) in dichloromethane (concentration 5 × 10^−5^ M).

UV-Vis electronic absorption spectra are shown in Figure 2 (continuous lines) together with the normalized fluorescence spectra. The absorption spectrum of compound **1** shows a maximum at λ = 482 nm (ε = 34,300 M^−1^cm^−1^) while the fluorescence band presents a peak at 534 nm. Both absorption and fluorescence maxima are very close to those reported for the same molecule in acetonitrile (Ragab et al., 2016), and they are very similar to those reported for the parent compound, diethylamino-NBD (**4**) (Fery-Forgues et al., 1993). The photophysical properties of NBD derivatives in different solvents have been investigated by Lopez and coworkers who reported for **4** in dichloromethane an absorption and fluorescence band with maxima at 482 and 534 nm, respectively (Fery-Forgues et al., 1993). These bands are attributed to an electronic transition with charge transfer (CT) character, the amino group acting as the electron donor and the nitro group as the acceptor. Because of its nature, the CT bands are affected both by the polarity of the environment (e.g., the solvent) and of substituents. Considering the similarity between NBD-amine and **1**, we can conclude that the triphenylphosphazene group and the diethylamino group have a very similar electronic effect on the nitro-aromatic system. Moreover, the fluorescence QY = 0.50 and the excited state lifetime τ = 5.7 ns of 1 in CH_2_Cl_2_ are very similar to those of NBD.

The introduction of the methyl group on the phenyl substituent to give **2** produces a shift of the absorption maximum to 488 nm and of the fluorescence to 524 nm. It is interesting observing that going from **1** to **2**, only a very small decrease of the energy of the lowest singlet excited state is observed, from 2.44 eV for **1** to 2.45 eV for **2**, but a relevant decrease of the Stokes shift from 0.26 to 0.18 eV occurs. This indicates that the effect of the methylation is to increase the hindrance of the bulky triphenylphosphazene substituents on the phosphorous atom and hence it reduces the degree of conformational reorganization of the excited states which produces the Stokes shift. The electronic effect of the weak electron donor methyl group, on the other hand, is marginal and since the electronic transition has a strong charge-transfer character, no relevant difference in the electronic transition is observed. The rigidification effect due to the bulkier substituents also affects the fluorescence QY and the excited state lifetime that rises, with respect to **1**, to QY = 0.65 and τ = 7.7 ns in **2**.

On the contrary, the substitution of each of the three phenyl rings with three strong electron donor methoxy groups to give **3** produces a large hypsochromic shift both of the absorption band, λ = 524 nm (ε = 26,200 M^−1^cm^−1^) and of the fluorescence maximum λ_em_ = 555 nm, corresponding to a decrease of the energy of the transition to 2.30 eV. The further increase of the hindrance of the phosphazene substituents in **3** with respect to **2** leads to a decrease of the Stokes shift to 0.13 eV. On the other hand the presence of the electron rich trimethoxyphenyl group causes a decrease of the fluorescence QY to 0.09 and of the excited state lifetime to τ = 4.2 ns

### Preparation and Characterization of the NPs

Dye molecules **1**, **2**, and **3** are water insoluble and nanoparticles (NPs) were prepared by nanoprecipitation (Reisch and Klymchenko, 2016). A small volume (10 μL) of a THF solution of **1**, **2** or **3** (2 mg/ml) and the surfactant Pluronic F127 (20 mg/mL) was rapidly injected into 2.5 mL of Millipore water under vigorous stirring. The reaction vial was kept open to atmospheric air in order to allow complete evaporation of the organic solvent. After 2 h of stirring, a precipitate was formed in the case of compound **1** while transparent, colored suspensions were obtained for samples containing **2** and **3**.

**Figure 3:**
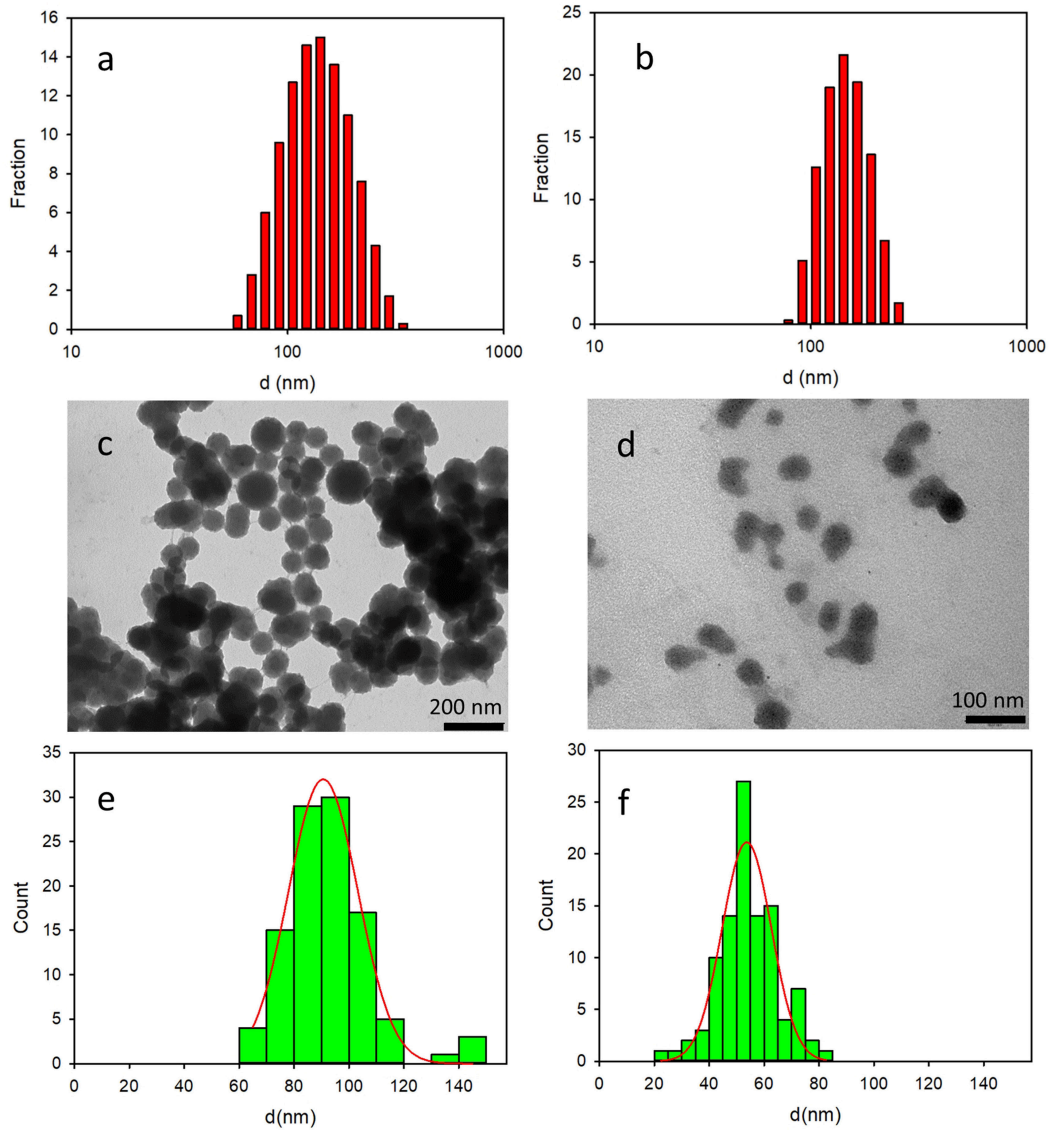
Top: size distribution obtained by DLS analysis of 2NP **(a)** and 3NP **(b)**. Center: representative TEM images of 2NP **(c)** and 3NP **(d)**. Bottom: size distribution resulting from the analysis of the TEM images of 2NP **(e)** and 3NP **(f)**.

The formation of the NP constituted by **2** and **3** was demonstrated by dynamic light scattering (DLS), transmission electron microscopy (TEM) and fluorescence microscopy (FM). In particular, DLS measurements showed the presence of a quite monodisperse single population of NPs both in the sample containing **2** (**2NP**, d = 128 nm, PdI = 0.11) and **3** (**3NP**, d = 140, PdI = 0.06). The size distribution of the two samples is shown in Figures 3a,b, respectively. DLS measurements were performed after dilution of the NPs suspension in phosphate-buffered saline (PBS) solution (1:50, vol:vol). After such a dilution, the concentration of the surfactant Pluronic F127 was 0.13 μM, hence more than three order of magnitude below the critical micelles concentration (cmc = 0.3 mM at r.t.) (Rampazzo et al., 2011). In order to exclude the formation of NPs constituted by the surfactant (micelles that, on the other hand, have been reported to show size of tens of nm), a blank sample was prepared following the same procedure used for **2NP** and **3NP**. No relevant scattering signal was detected in the case of the blank samples, confirming the absence of NPs.

The size distribution of **2NP** and **3NP** was also investigated by TEM: representative images of the two samples are shown in Figures 3c,d, respectively while the histogram obtained by measuring the size of the NP in the images with the software Image J are shown in Figures 3e,f. Fitting the data with a Gaussian model, we obtained the NP size: 91 ± 13 and 54 ± 9 for **2NP** and **3NP**, respectively. While the diameter measured for **2NP** by TEM was consistent with the hydrodynamic diameter measured by DLS, a significant difference was observed in the case of **3NP**. The larger hydrodynamic diameter measured by DLS for this latter sample revealed a partial aggregation of **3NP** in water.

The photophysical characterization of **2NP** and **3NP** was performed in PBS, results are summarized in Table 1. Since it was not possible to compare the properties of **2NP** and **3NP** with the molecular components **2** and **3** in aqueous medium because of their insolubility, we compared them to the parent compound diethylamino-NBD (**4**), as shown in Table 1.

**Figure 4:**
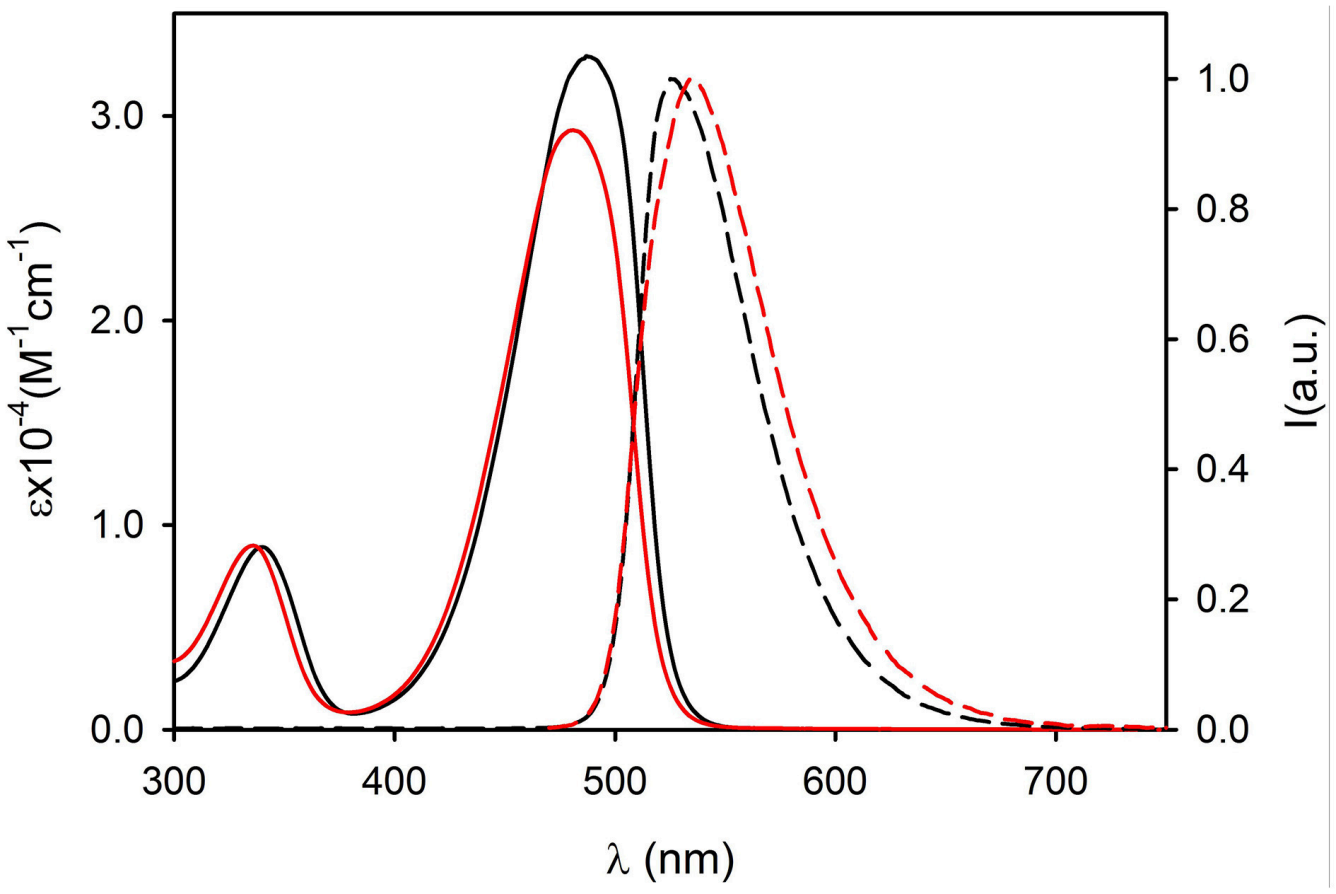
Absorption (continuous lines) and fluorescence spectra (dashed lines, λ_exc_ = 450 nm) of compound 2 (black, dichloromethane) and of 2NP (red, PBS).

**Figure 5:**
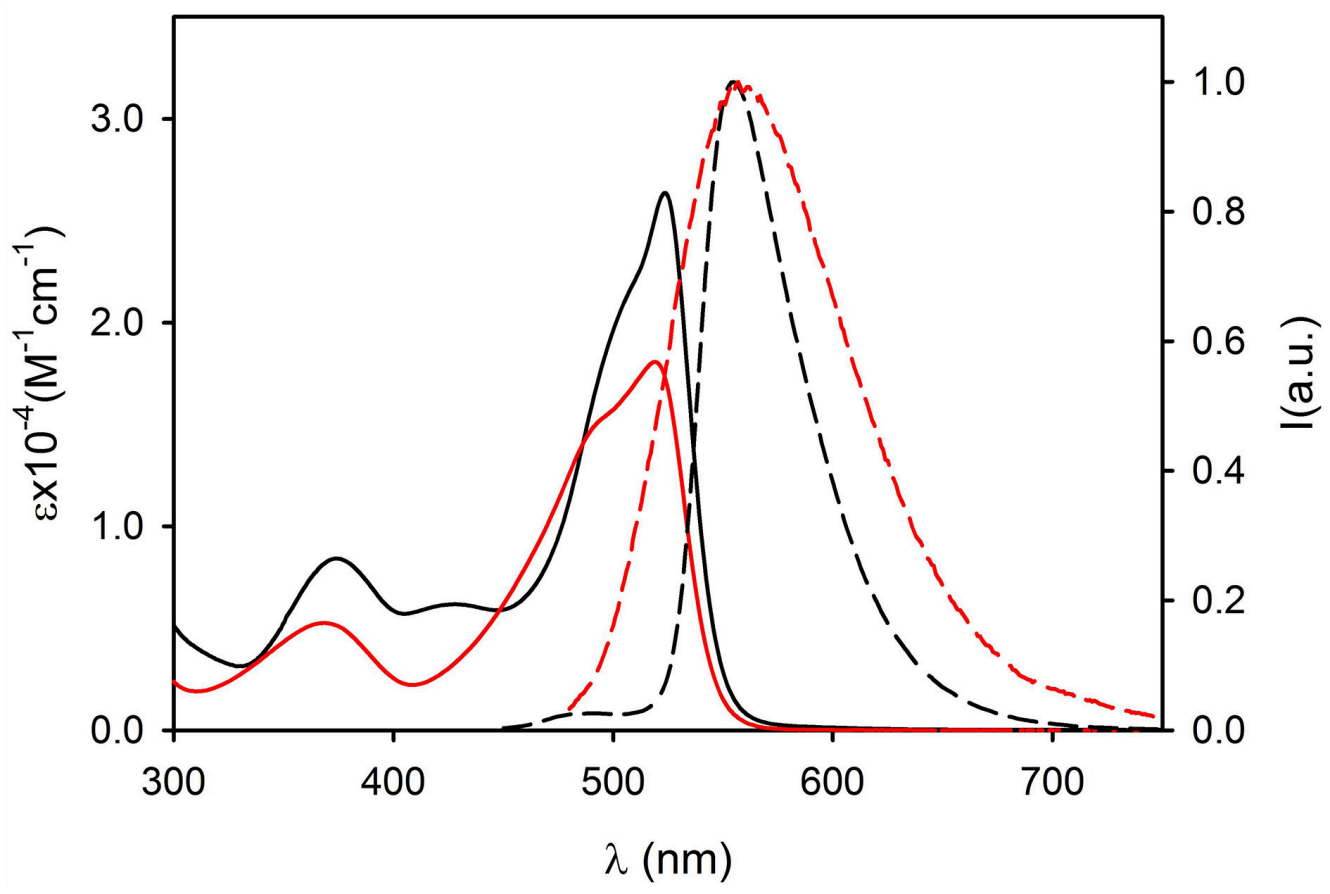
Absorption (continuous lines) and fluorescence spectra (dashed lines, λ_exc_ = 490 nm) of compound 3 (black, dichloromethane) and of 3NP (red, PBS).

The UV vis absorption spectra of **2NP** and **3NP** are shown in Figures 4, 5, respectively. The molar absorption coefficient (ε) was calculated for the molecules **2** and **3** in the NP considering their average concentration. Figures 4, 5 clearly show that the aggregation in the NP has only a minor effect on the absorption properties of the dye molecules that undergo only a modest hypsochromic shift and a moderate decrease of ε.

On the contrary, NP formation had a very different effect on the fluorescence properties of **2** and **3**. In particular, **2** maintains in the NPs an acceptable quantum yield (QY = 0.31) while **3** undergoes strong aggregation induced quenching (QY = 0.01). This observation suggests that the introduction of methyl on the bulky tri(phenyl)phosphazene group is suitable to decrease the intermolecular electronic interactions in the NPs by reducing the overlap of the molecular orbitals of the fluorescent NBD units. Nevertheless, the presence of electron donating tri-methoxyphenyl groups is known to cause fluorescence quenching because of the formation of charge-transfer non-fluorescent excited states (Shukla and Wan, 1993). Fluorescence anisotropy measurements demonstrated that the quenching effect is enhanced by excitation energy migration inside the NPs (Bonacchi et al., 2008; Jiang and McNeill, 2017). Both **2** and **3** in fact showed in a high viscosity medium like propylene glycol, quite a high value of fluorescence anisotropy *r* (*r* = 0.21 for **2** and *r* = 0.23 for **3** at r.t.). On the contrary, the two fluorophore immobilized in the NPs showed a fluorescence anisotropy which is zero both for **2NP** and **3NP**. The complete depolarization observed in the NP is in contrast with the lack of rotational freedom of the fluorophores in the nanostructures and can be explained only by considering a fast depolarization involving the homo-energy transfer processes (Genovese et al., 2013).

Time resolved fluorescence measurements (time correlated single photon counting, TCSPC) showed that the spectral changes observed upon NP formation were due to the presence, in the NPs, of populations of fluorophores experiencing different environments. Tri-exponential decays were observed both in the case of **2NP** (τ_1_ = 0.46 ns, B_1_ = 3,196, τ_2_ = 1.45, B_2_ = 1,085, τ_3_ = 6.16, B_3_ = 44) and **3NP** (τ_1_ = 0.62 ns, B_1_ = 4,132, τ_2_ = 2.44, B_2_ = 3,724, τ_3_ = 7.81, B_3_ = 1,559). From these data the average excited lifetime was calculated to be < τ> = 2.53 ns and < τ> = 0.77 ns for **2NP** and **3NP**, respectively. This result is in agreement with the low fluorescence QY measured for **3NP**.

In order to evaluate the order of magnitude of the fluorescence brightness of **2NP** and **3NP**, the number of molecules per particles was estimated as reported in Table 1. This number was calculated considering the molar volume of **2** and **3** (molecular volume of **1**, **2**, and **3** were calculated to be 338.0, 380.9, and 533.6 Å3, respectively) and the hydrodynamic diameter of the NPs.^1^

(1) $$\begin{array}{l} V=\frac{\pi }{6}d^{3}N \end{array}$$

Molar volume for **2NP** and **3NP** were 2.4 × 10^5^ and 5.0 × 10^4^ L mol^−1^ while molar volume for **2** and **3** were 0.23 Lmol^−1^ and 0.32 L mol^−1^, respectively. Hence **2NP** and **3NP** contain about 1.1 × 10^6^ molecules and 1.6 × 10^5^ dye molecules, respectively. Considering these values, the brightness of **2NP** results to be as high as ~10^10^M^−1^cm^−1^ while **3NP** show a brightness which is almost two orders of magnitude lower than **2NP**.

### Size Characterization of 2NP by Fluorescence Optical Tracking

**Figure 6:**
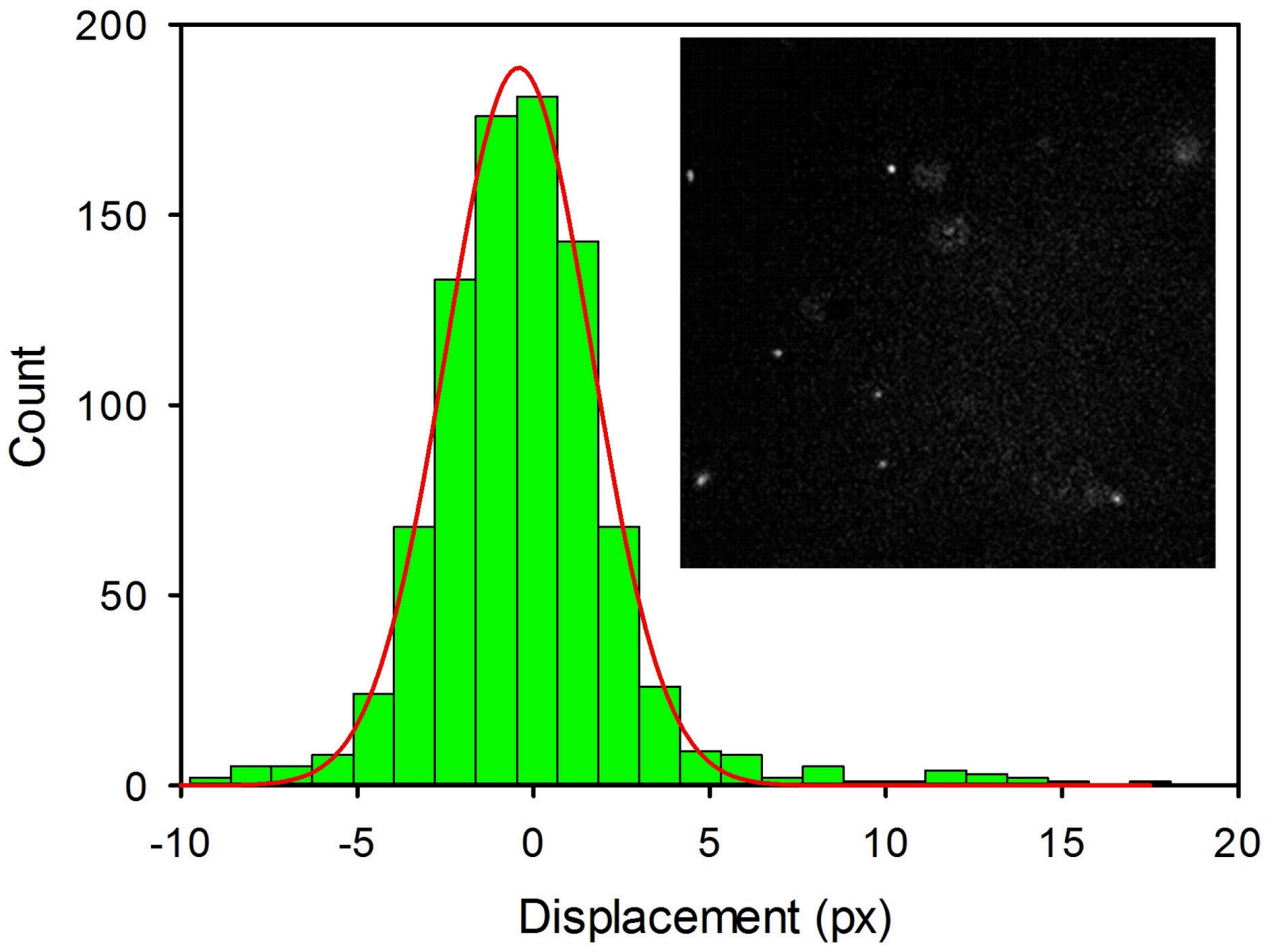
Histogram of the linear displacements measured for 2NP in PBS by fluorescence microscopy for a time interval Δt = 10 ms. Images were acquired in time-lapse mode (4,000 frames) with an EMCCD Camera and processed with Image J (Plug-in MOSAIC). Each pixel corresponds to 0.16 μm. A representative image of the NP (bright white spots) is shown in the inset.

Thanks to their outstanding brightness, **2NP** could be detected, in suspension, as single bright spots in a conventional fluorescence microscope. Using an acquisition time as short as 10 ms, the NP appeared motionless as shown in Figure 6 (inset). Thermal motions of the NP were clearly observed by time lapsed acquisition (4,000 frames). The resulting movies were analyzed to measure the linear displacements of the NPs using the plugin MOSAIC for Image J (Sbalzarini and Koumoutsakos, 2005; Chenouard et al., 2014). The displacements were then plotted in a histogram as shown in Figure 6. We would like to stress that an analogous experiment was performed for a **3NP** sample and no emissive spots attributable to NP diffusion could be observed. This demonstrated that these NPs were not bright enough to be detectable as individual objects by fluorescence microscopy. Moreover, by comparing two samples of **2NP** and **3NP** with the same concentration (8 μg/ml), the average intensity measured within a frame in the case of **2NP** was more than 2 orders of magnitude higher than the one measured for **3NP**.

The diffusion coefficient of **2NP** was calculated by tracking the fluorescent NP via fluorescence microscopy considering the following equations:

(2) $$\begin{array}{r} P\left(x,t\right)=\frac{1}{\sqrt{4\text{π}Dt}}\exp\left(-\frac{{\left(x-x_{0}\right)}^{2}}{4Dt}\right) \end{array}$$

Where P is the probability of observing a displacement of an NP from the position x_0_ to the position × after a time delay t and D is the diffusion coefficient of the NP that, in a spherical approximation, is dependent on the diameter of the NP according to the Stokes-Einstein equation:

(3) $$\begin{array}{r} D=\frac{kT}{3\pi \eta d} \end{array}$$

Where k is the Boltzmann constant, T the temperature and η is the viscosity of the medium. Image sequences were processed to acquire the positions of the NP in each frame and to identify individual NP movements. The trajectories of the NPs were used to get the displacement (in pixels, where a pixel corresponds to 0.16 μm) of the NP in the frame acquisition time interval (t = 1.0 × 10^−2^ s). Data represented in the histogram were fitted with a Gaussian model, as shown in Figure 6, to obtain the diffusion coefficient D = 2.65 × 10^−12^ m^2^s^−1^; a value that corresponds to NPs with an average diameter of 160 nm in good agreement with the DLS analysis.

### Biological Experiments

**Figure 7:**
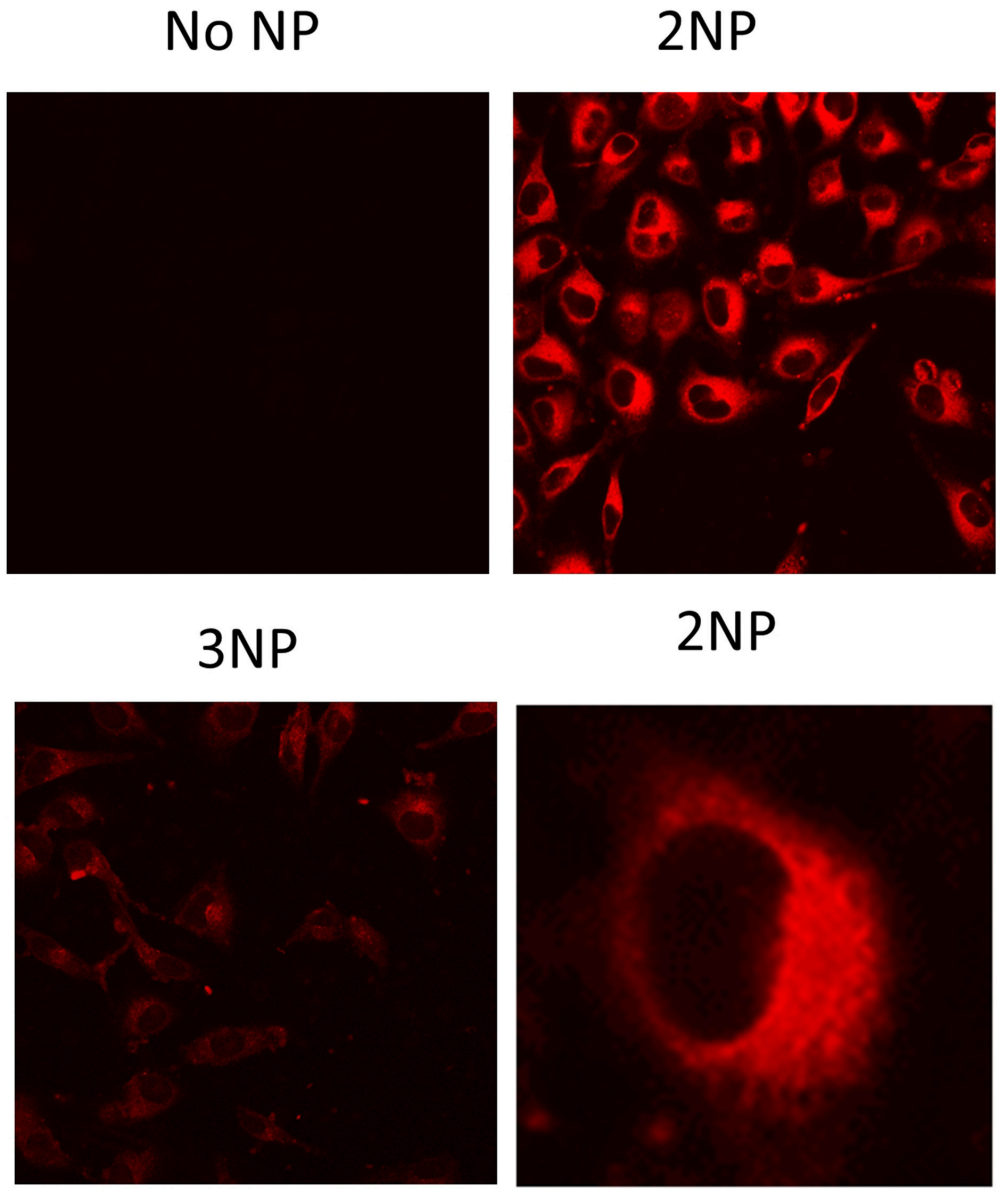
Fluorescence signal of NPs in cells. HeLa cells grown on glass cover-slips were incubated for 20 h with no NPs (medium alone), 2NP or 3NP, as indicated and analyzed by fluorescence confocal microscopy with the same instrumental setting for comparison. Representative images are shown. In the case of 2NP cell signal distribution details are shown at higher magnification. Bars are the standard deviations of the means.

**Figure 8:**
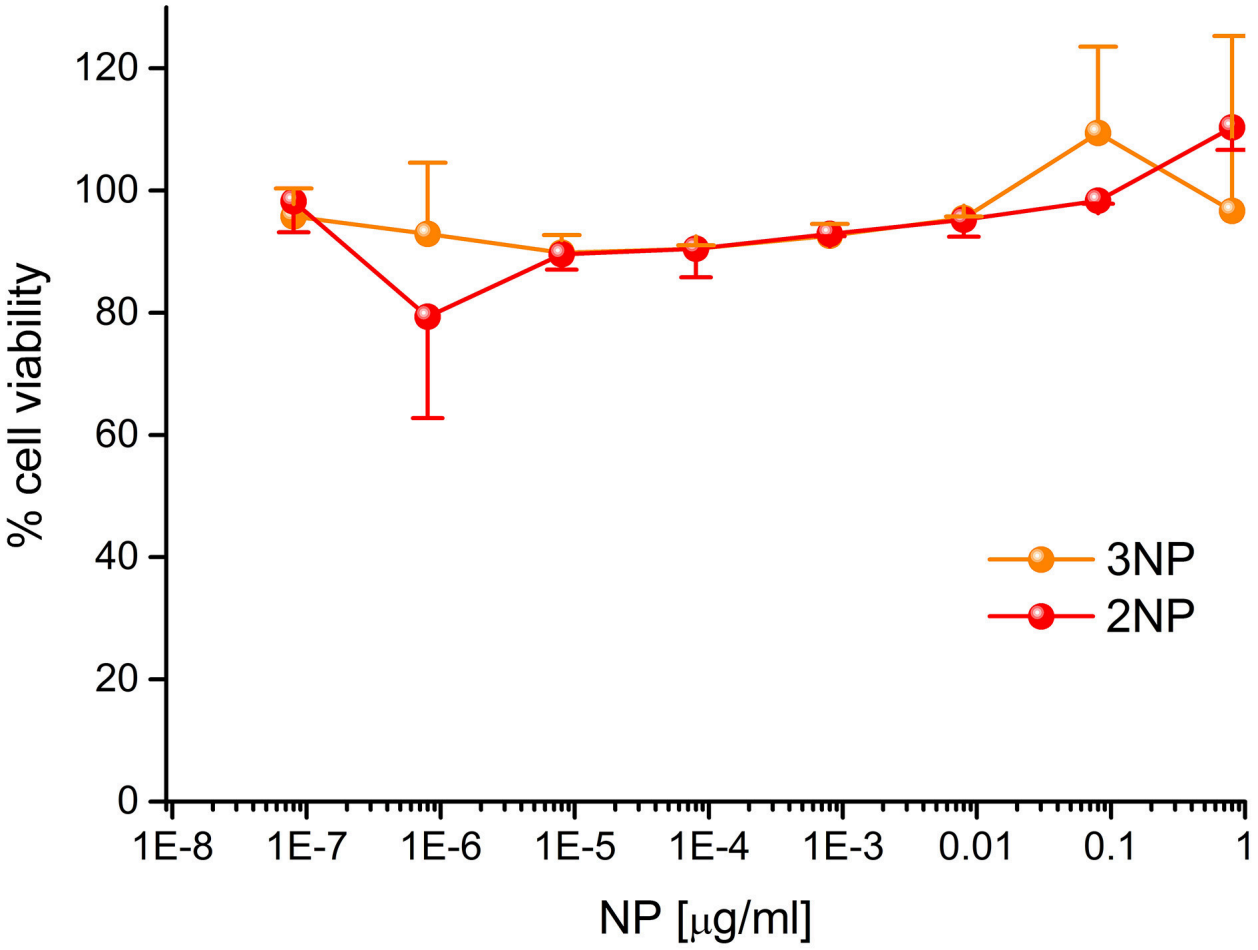
Cytotoxicity of 2NPs and 3NPs. HeLa cells were incubated for 20 h with the indicated doses of NPs in DMEM at 37°C and subjected to MTS assay. Data, expressed as % of control (no NP) samples, are mean ± SE (N=3).

To demonstrate their efficacy as a fluorescent probe, **2NP** and **3NP** were incubated with HeLa cells at the relatively low dose of 80 ng/ml at 37°C. After confocal microscopy analysis of 20 h, cells (Figure 7) revealed an intense structured **2NP** signal within the cell cytoplasm, suggesting endosomal NP internalization, although cytoplasmatic internalization cannot be completely ruled out. As expected, based on their weak intrinsic fluorescence, **3NP** cellular signal was much weaker. MTS assays showed that both **2NP** and **3NP** are devoid of the toxic effects on HeLa cells of up to 1 μg/ml (Figure 8).

## Conclusions

Summarizing the results so far discussed, we found that molecule **1** does not form stable NP upon nanoprecipitation in our experimental conditions. On the contrary, nanoprecipitation of molecules **2** and **3** leads to NP formation. However, the quantum yield of molecule **3**, which is already low in the non-aggregated form, further decreases upon assembly in the NP. As a result, the brightness of **3NP** is about two orders of magnitude lower than the one of **2NP**. Such a difference is so relevant that while **2NP** can be clearly tracked at the single NP level in solution at very low concentration by fluorescence microscopy, **3NP** cannot be observed with the same technique. Most interestingly, the brightness of **2NP** could be estimated to be about six orders of magnitude higher than an NBD water soluble derivative used as reference. These results demonstrate that rational design of the molecular precursor is fundamental for producing stable and strongly bright nanoparticles by self-assembly. Cellular experiments proved that **2NPs** are suitable to be used as fluorescent contrast agents for bioimaging, also thanks to their good biocompatibility. We believe that our approach can be extended to other molecules and surfactants in order to tune the excitation/emission wavelength as well as the NP size.

## Experimental Section

General: Solvents were purified by standard methods. All the reagents used were purchased by Sigma-Aldrich and used as received.

TLC analyses were performed using Merck 60 F254 precoated silica gel glass plates. Column chromatography was carried out on Macherey-Nagel silica gel 60 (70–230 mesh).

NMR spectra were recorded using a Bruker AV 500 spectrometer operating at 500 MHz for 1H, 125.8 MHz for 13C. Chemical shifts are reported relative to internal Me4Si. Multiplicity is given as follow: s = singlet, d = doublet, t = triplet, q = quartet, qn = quintet, m = multiplet, br = broad peak.

ESI-MS mass spectra were obtained with an Agilent Technologies LC/MSD Trap SL mass spectrometer. EI/MS spectra were obtained with an Agilent Technologies 6850-5973 GS/MS.

### Synthesis of 4-Azido-7-Nitrobenzofurazan (NBD-N_3_)

Into a flask, covered with an aluminum foil and containing NaN_3_ (0.350 g, 5.39 mM) dissolved in a EtOH/H_2_O mixture (1:1 v/v, 20 mL), a solution of NBD-Cl (0.5 g, 2.505 mM) in EtOH (40 mL) was added dropwise within a 1 h period. The reaction was left stirring for 6 h at RT. Subsequently, the solvent was removed under reduced pressure and crude was purified via column chromatography on silica gel using petroleum ether/AcOEt 4:6 as eluent. The product **1** was obtained as an orange solid in 90% yield.

^1^H NMR (300 MHz, d_6_-DMSO), δ: 8.70 (d, *J* =5.3 Hz, 1H), 6.40 (d, *J* = 5.3 Hz, 1H).

ESI/MS, m/z: 207.8 (15%, M+H^+^), 178.8 (100%, M-N_2_+H^+^).

EI/MS, m/z: 207 (100%, M^+^), 180, 150, 133, 120, 104, 92, 77, 64, 52.

### Synthesis of 4-Triarylphosphazo-7-Nitrobenzofurazan (1-3)

Into a flask, covered with aluminum foil and containing **1** (0.1 g, 0.485 mM) and the desired phosphine (3 eq.) dissolved in THF (8 mL), H_2_O (2.6 mL, 145.553 mM) was added at once and left to react overnight under stirring. Subsequently, the formed solid was filtered-off, washed with cold THF and dried giving the final product as an orange solid with quantitative yield.

#### 4-Triphenylphosphazo-7-Nitrobenzofurazan (1)

^1^H NMR (300 MHz, CDCl_3_) δ 8.19 (d, *J* = 8.3 Hz, 1H), 7.74 (m, *J* = 13.0, 10.7, 4.7 Hz, 6H), 7.65–7.56 (m, 3H), 7.54–7.37 (m, 7H), 6.56 (d, *J* = 8.5 Hz, 1H).

^31^P NMR (202 MHz, CDCl_3_) δ 14.01.

^13^C NMR (126 MHz, CDCl_3_) δ 137.04, 134.06, 134.02, 133.06, 132.92, 130.21, 130.04, 126.84, 125.51, 40.86, 40.58, 40.30, 40.02, 39.74, 39.47, 39.19.

ESI-MS, m/z: 425.2 (100%, M+H^+^).

#### 4-Tri-(2-Methylphenyl)Phosphazo-7-Nitrobenzofurazan (2)

^1^H NMR (500 MHz, DMSO-*d*_6_) δ 8.20 (d, *J* = 8.8 Hz, 1H), 7.71 (t, *J* = 7.4 Hz, 3H), 7.58–7.46 (m, 10H), 5.50 (d, *J* = 8.9 Hz, 1H), 2.20 (s, 9H).

^31^P NMR (202 MHz, DMSO-*d*_6_) δ 21.31.

^13^C NMR (126 MHz, DMSO-*d*6) δ 154.93, 150.91, 143.15, 143.08, 137.24, 134.40, 134.32, 134.09, 133.98, 133.49, 133.40, 127.61, 127.51, 123.92, 123.15, 121.57, 117.25, 109.16, 109.06, 31.16, 30.06.

ESI-MS, m/z: 483.2 (100%, M+H^+^), 505.1 (10%, M+Na^+^).

#### 4-Tri-(2,4,6-Triemthoxyphenyl)Phosphazo-7-Nitrobenzofurazan (3)

^1^H NMR (500 MHz, Acetone-*d*6) δ 8.54 (d, *J* = 8.6 Hz, 1H), 8.05 (d, *J* = 9.4 Hz, 1H), 7.90 (s, 1H), 6.25 (d, *J* = 4.7 Hz, 6H), 5.83 (d, *J* = 8.4 Hz, 1H), 3.88 (s, 10H), 3.60 (s, 27H).

^31^P NMR (202 MHz, Acetone-*d*_6_) δ 3.03.

^13^C NMR (126 MHz, Acetone-*d*_6_) δ 167.49, 163.92, 142.36, 140.66, 140.53, 135.71, 129.25, 125.86, 118.39, 96.10, 56.14, 55.65.

ESI-MS, m/z: 711.2 (100%, M+H^+^).

#### Absorption and Fluorescence Spectra

UV-VIS absorption spectra were recorded at 25°C by means of Cary 300 UV-Vis spectrophotometer (Agilent Technologies).

#### Steady State Fluorescence Spectra

The fluorescence spectra were recorded with a Horiba Fluoromax-4 spectrofluorimeter and with an Edinburgh FLS920 fluorimeter equipped with a photomultiplier Hamamatsu R928P. Quartz cuvettes with optical path length of 1 cm were used for both absorbance and emission measurements.

#### Excited State Lifetimes

Excited state lifetime was measured with an Edinburgh FLS920 fluorimeter equipped with an electronic card for time correlated single photon counting TCSPC900. The kinetic tracks were fitted with a tri-exponential model: $I\left(t\right)=A+B_{1}e^{-t/\tau_{1}}+B_{2}e^{-t/\tau_{2}}{+\;B}_{3}e^{-t/\tau_{3}}$ with the software package FAST.

#### Fluorescence Anisotropy Spectra

All fluorescence anisotropy measurements were performed on an Edinburgh FLS920 equipped with Glan-Thompson polarizers. Anisotropy measurements were collected using an L-format configuration, and all data were corrected for polarization bias using the G-factor.

In particular four different spectra were acquired for each sample combining different orientations of the excitation and emission polarizers: I_VV_, I_VH_, I_HH_, I_HV_ (where V stands for vertical and H for horizontal with respect to the plane including the excitation beam and the detection direction; and the first subscript refers to the excitation and the second subscript refers to the emission). The spectra were used to calculate the G-factor and the anisotropy r: G = I_HV_/I_HH_ and r = I_VV_-GI_VH_/I_VV_+2GI_VH_.

#### Epifluorescence Microscopy

The fluorescence images were obtained with an inverted microscope (Olympus IX71) equipped with a Xenon lamp for excitation. Excitation, dichroic and emission filters were purchased from Chroma and Thorlabs. Excitation filter: 475 ± 17.5 nm; emission filter: 530 ± 21.5 nm; dichroic Reflection/Transmission): 470–490/508–675 nm. Fluorescence images were acquired with an Electron Multiplying Charge Coupled Device EMCCD Camera (Princeton Instruments, Photon Max 512). Acquisition time was 30 ms per frame at the maximum amplification gain using a 100x oil immersion objective for fluorescence (Olympus UPLFLN100XO2).

#### Particle Tracking

Trajectories were tracked by analyzing sequences of images acquired with an integration time τ of 10 ms per frame. The particles were localized and tracked by using the plug-in MOSAIC for the software ImageJ (Sbalzarini and Koumoutsakos, 2005; Chenouard et al., 2014). The displacement distribution was processed with the software Sigmaplot to obtain histograms that were fitted with Gaussian peaks. The Stokes-Einstein equation was used to obtain the particle diameter *d*.

#### Dynamic Light Scattering

Light Scattering measurements were performed using a Malvern Nano ZS instrument equipped with a 633 nm laser diode. Samples were housed in disposable polystyrene cuvettes of 1 cm optical path length. DLS measurements were performed after dilution of the NPs suspension in phosphate-buffered saline (PBS) solution (1:50, vol:vol).

#### Transmission Electron Microscopy

A Philips CM 100 transmission electron microscope operating at 80 kV was used. For TEM investigations, a 3.05 mm copper grid (400 mesh) covered by a Formvar support film was dried up under vacuum after deposition of a drop of nanoparticles solution.

#### Cellular Experiments

HeLa cells were maintained in a DMEM medium (Gibco) supplemented with 10% FCS (Euroclone) and antibiotics (penicillin and streptomycin, Invitrogen) at 37°C in a humidified atmosphere containing 5% (v/v) CO_2_; cells were split every 2–3 days. For MTS cytotoxicity assay, cells (1 × 10^4^ cells) were plated onto a 96-well culture plate the day before the experiment. Cells were then incubated for 20 h with NPs at different concentrations in DMEM, added with 10% FCS. Cellular mitochondrial activity (indicator of cellular viability) was evaluated by MTS assay (Promega) according to the instruction manual. For the MTS test N = 3 independent experiments were run in triplicate. *t* test (significativity *p* < 0, 05) were performed, but differences compared to control (no particles) were always not significant (*p* > 0, 05). For the assessment of intracellular distribution of NPs, cells (1 × 10^5^) were seeded on cover glasses and after 24 h they were incubated for 20 h at 37°C with NPs, washed with PBS and directly analyzed by confocal microscopy (Leica SP2). Images were processed using ImageJ software.

## Data Availability

All datasets generated for this study are included in the manuscript and/or the supplementary files.

## Author Contributions

MM designed the synthesis of the NPs and supervised their preparation and characterization. FM and JT synthesized molecules 1, 2, and 3. VC prepared and characterized the NP. AC performed the photophysical characterization of molecules. RT and EP performed the cellular experiments.

### Conflict of Interest Statement

The authors declare that the research was conducted in the absence of any commercial or financial relationships that could be construed as a potential conflict of interest.

## Footnotes

^1^http://www.scfbio-iitd.res.in/software/drugdesign/VolumeCalculator.jsp.
